# Australian Notes

**Published:** 1919-11-29

**Authors:** 


### AUSTRALIAN NOTES.

### Appointments to University College (London).

Two graduates from the University of Sydney have been appointed to University College (London). Professor Elliot Smith has succeeded Professor Thane in the Chair of Anatomy, after eight years in the same position in the University of Manchester. Professor W. D. Hill was a lecturer at Sydney for twelve years, and is now professor of zoology at University College, in succession to Sir Ray Lankester. These two have collaborated in valuable work upon the platypus, the unique Australian animal that is sometimes described as the "missing link."

### Major Sydney Wentworth Patterson.

The first director of the "Walter and Eliza Hall Institute of Research in Pathology and Medicine," in the person of Major Sydney Wentworth Patterson, graduated M.B.(Melb.) in 1904 and M.D. in 1907. He took first-class honours in chemistry, physiology, and medicine, and was resident medical officer at Melbourne Hospital and the Children's Hospital. Proceeding to England, he was elected a Beit Memorial Research Fellow, and in 1917 his researches won him the triennial Schafer Prize, and the University of London conferred on him the degree of Doctor of Science in Physiology.

